# Ectopic Expression of the *Allium cepa 1-SST* Gene in Cotton Improves Drought Tolerance and Yield Under Drought Stress in the Field

**DOI:** 10.3389/fpls.2021.783134

**Published:** 2022-01-12

**Authors:** RuiNa Liu, TianQi Jiao, ZeXing Zhang, Zhang Yao, ZhongQing Li, Saisai Wang, Hongliang Xin, YuXia Li, AiYing Wang, JianBo Zhu

**Affiliations:** ^1^The Key Laboratory of Agricultural Biotechnology, College of Life Sciences, Shihezi University, Shihezi, China; ^2^Woda Agricultural Technology Co., Ltd, Shihezi, China

**Keywords:** cotton, ectopic expression of *1-SST*, drought, yield, relative water content

## Abstract

In some plants, sucrose: sucrose 1-fructosyltransferase (1-SST) is the first irreversible key enzyme in fructan biosynthesis. Studies have shown that fructan accumulation enhances abiotic stress tolerance of plants. To investigate the role of *1-SST* in drought stress responses, a total of 37 cotton plants expressing a *1-SST* gene from *Allium cepa* were developed by *Agrobacterium*-mediated transformation. Under drought stress in the field, compared with wild-type, ectopic expression of *Ac1-SST* in cotton resulted in significantly higher soluble sugars (especially 1-kestose), proline and relative water contents, as well as decreased malondialdehyde content, which contributed to maintaining intracellular osmoregulation and reducing membrane damage. In addition, ectopic expression of *Ac1-SST* in cotton significantly improved the photosynthesis rate, performance of PSII (including Pn, Fv/Fm, WUE, ΦPSII, and PI_total_) and plant growth under drought stress. Furthermore, compared with the wild-type, under the droughted field, the yield loss per square meter of transgenic cotton was reduced by an average of 20.9% over two consecutive years. Our results indicate that the *Ac1-SST* gene can be used to improve drought tolerance and yield of cotton varieties, and might also be a promising drought-resistant gene for improving other crop varieties.

## Introduction

Fructans are synthesized in bacteria, fungi, and higher plants. Apart from their role as a carbon store in higher plants (Van den Ende et al., 2011), fructans have many important physiological functions (Vijn and Smeekens, 1999) such as protecting plants against water deficit caused by drought or low temperature (Hendry, 1993; Pilon-Smits et al., 1995). About 15% of the angiosperm flora in the world, which are mainly distributed in Compositae (dicotyledons), Poaceae and Liliaceae (monocotyledons), store fructans (Hendry, 1993). In higher plants, sucrose: sucrose 1-fructosyltransferase (1-SST) is considered as a key enzyme in fructan biosynthesis, where it catalyzes the first step of fructan biosynthesis. Specifically, 1-SST is responsible for transferring the fructosyl moiety from one sucrose molecule to another to form a 1-kestose and release a glucose molecule, via the irreversible reaction: $G\,F+G\,F\overset{1-S\,S\,T}{⟶}G\,F_{2}+G$ (Edelman and Jefford, 1968; Koops and Jonker, 1994; Lüscher et al., 1996; Van Den Ende et al., 1996). A recent study showed that overexpression of *1-SST* was essential for production of long-chain inulin in chicory (Maroufi et al., 2018). In addition, fructans, like starch and sucrose, are important stored forms of carbohydrate, and are also closely related to the regulation of plant carbon allocation and sucrose-pool (Pollock, 1986; Nelson and Spollen, 1987). However, the most obvious differences between fructan and starch are mobility and solubility. In contrast to the largely insoluble and compact starch, fructan is soluble and osmotically active. Thus, fructans could play a role in osmotic adjustment through variation in the degree of polymerization of fructan pools. In general, fructans represent an evolutionary advantage for fructan accumulators by supporting efficient adaptation to environmental stresses (Hendry, 1993). Tognetti et al. (1989) found that the content of fructans was associated with cold resistance. Further studies have confirmed those results (Van Den Ende and Van Laere, 1996; Vágújfalvi et al., 1999; Savitch et al., 2000; Michiels et al., 2004). Fructan accumulation was also found to be associated with drought resistance, for example, the fructan concentration in the roots and leaves of drought stressed plants was ten times higher than those of the well-watered ones (De Roover et al., 2000). The drought resistant wheat cultivar LH7 had higher expression levels of the genes related to fructan biosynthesis and degradation during early and late stages of drought stress (Hou et al., 2018). A recent study also showed that fructans play a crucial role in the tolerance of wheat seedlings to drought stress (Nemati et al., 2018). All the above results show that fructans play an important role in the drought tolerance of plants. Moreover, fructans accumulation is also related to tolerance to salt, waterlogging and soil heavy metals (Frossard et al., 1989; Albrecht et al., 1997, 2004; Kerepesi et al., 1998; Kerepesi and Galiba, 2000; Van den Ende et al., 2001; Suárez-González et al., 2014; Vandoorne et al., 2014). In particular, upon exposure to abscisic acid (ABA), which is the hormone that is associated with some key responses of plants to stress, fructan fructosyltransferases and fructan hydrolase were co-induced in chicory plantlets (Wei et al., 2016, 2017).

Since the first plant fructosyltransferase cDNA was cloned in 1995 (Sprenger et al., 1995), more genes related to fructans metabolism from different plants have been studied. With the rapid development of biotechnology, research on the fructan metabolism genes has become more extensive (reviewed in detail by Cairns, 2003; Livingston et al., 2009). However, most of the reported studies were carried out on tobacco (Caimi et al., 1997; Vijn et al., 1998; Luscher et al., 2000; Li et al., 2007; Banguela et al., 2011; Pilon-smits et al., 2016), potato (Hellwege et al., 1997, 2000; Stoop et al., 2007), and sugar beet (Sévenier et al., 1998). Only few studies involved food crops (Kawakami et al., 2008) and none of them included cash crops such as cotton.

Cotton (*Gossypium hirsutum* L.) is grown for textile fiber and oilseed, and hence is a main economic crop that is well adapted for plantation in the tropical and temperate regions around the world. Compared with other crops such as rice and wheat, cotton is considered as a drought/salt-tolerant crop and its tolerance varies greatly among genotypes (Ashraf, 2002). Nonetheless, drought stress greatly affects cotton growth, yield as well as fiber quality (Niu et al., 2018; Hu et al., 2019). Therefore, cotton breeders have focused recently on developing varieties with higher yield and drought-tolerance.

While it has been convincingly shown that fructan accumulation can increase drought tolerance (Knipp and Honermeier, 2006; Livingston et al., 2009), less is known whether accumulation of fructans with low degrees of polymerization (FLDP) affects drought tolerance under typical agricultural settings. We aimed to improve drought tolerance in the allotetraploid cotton plants using ectopic expression of the *1-SST* gene, which encodes the key enzyme for fructan biosynthesis. In this study, *Allium cepa L. 1-SST* gene which has been proved to synthesize structurally defined 1-kestose (FLDP) molecules from sucrose (Vijn et al., 1998), was cloned and introduced into upland cotton through *Agrobacterium*-mediated transformation. We expected to find individual transgenic lines in which FLDP is more accumulated and that such change may lead to enhanced drought resistance without detrimental growth penalties. The transgenic lines were grown under field conditions and subjected to a selection of traditional breeding procedures. The best performing plants were selected for drought treatment. Here, we describe these plant lines and show that the enhanced soluble carbohydrate content in the transgenic lines depends on the presence of water limiting conditions. The results show that *Ac1-SST* transgenic cotton lines showed improved drought tolerance in the field and also indicate that *Ac1-SST* may be a candidate gene for crop improvement.

## Materials and Methods

### Construction of Plant Expression Vector and Transformation of Cotton

The gene sucrose: sucrose 1-fructosyltransferase (*Ac1-SST*) was amplified from onion using the cDNA synthesis approach. Total RNA was extracted from leaves of onion using TriZol reagent (Invitrogen, United States) according to the manufacturer’s protocol. The reverse transcription reaction mixture consisted of 12 μL of RNA, 1 μL of RNase inhibitor (10 U/μL), 0.5 μL of Olig(dT)_18_ (10 pmol/μL), 4 μL of 5x Buffer M-Mulv Reverse Transcriptase, 2.5 μL of dNTP (2.5 mmol/L) and 2 μL of M-Mulv Reverse transcriptase enzyme (200 U/μL). The mixture was gently mixed and incubated at 42°C for 1 h, 72°C for 10 min and 4°C for 10 min. The cDNA product was used as a template to amplify the *Ac1-SST* gene with the specific primers listed in Table 1, designed based on the full sequence of *Ac1-SST* in the GeneBank database. The PCR reactions consisted of pre-denaturation at 94°C for 10 min, followed by 30 cycles of denaturation at 94°C for 30 s, annealing at 56°C for 30 s, elongation at 72°C for 1 min, and a final elongation step at 72°C for 7 min. The amplified *1-SST* was verified by running on 1% agarose gel. The target PCR fragment was ligated into the pGEM-T Easy Vector (Tiangen) for sequencing.

**TABLE 1:** Primers used in this study.

Primer name	Primer sequence
1-SST_F	5′—TCTAGA (*Xba*I) CCATGGAATCCAGAGAGATCGAG—3′
1-SST_R	5′—GAGCTC (*Sac*I) TGAGCACCTAACCAAACAACACA—3′
UBQ_F	5′—AGAGGTCGAGTCTTCGGACACC—3′
UBQ_R	5′—TGCTTGATCTTCTTGGGCTTGG—3′
qRT 1-SST_F	5′—ATCGGGAACGGGCTTGAAAT—3′
qRT 1-SST _R	5′—TACCTCAAGCCGACACCAAC—3′

*1-SST, primer for Ac1-SST gene; UBQ, primer for qRT-PCR of cotton internal reference; qRT 1-SST, primer for qRT-PCR of Ac1-SST gene. The underlined segments are the enzyme cleavage site.*

The pGEM-*1-SST* recombinant plasmid was digested using the restriction enzymes *Xba*I and *Sac*I and ligated into the binary expression vector pBI121 under control of the cauliflower mosaic virus 35S promoter (CaMv 35S) using T_4_ DNA ligase enzyme. Plasmids identified as positive by PCR and enzyme digestion were transformed into *Agrobacterium* strain GV3101 by the freeze-thaw method and used for upland cotton (*Gossypium hirsutum*) R15 transformation.

The genetic transformation of cotton was carried out as previously described (Liu et al., 2019). Briefly, the hypocotyls of surface-sterilized cotton seedlings grown for 7 days were cut into 1 cm segments, and the injured samples were infected with the *Agrobacterium tumefaciens* carrying the pBI121-*Ac1-SST* plasmid for 7-10 min. After two days of co-cultivation in darkness, the hypocotyls were transferred to different differentiation media until embryogenic calli, immature embryos, mature embryos and seedlings developed. All putative T_0_ tissue cultured seedlings were first grafted onto stems of the upland cotton R15 to ensure survival. Then, they were screened for resistance to kanamycin (7,000 ppm) and the presence of the target gene was verified using genomic PCR. All PCR-positive plants were grown until they set seeds. The T_2_-T_6_ *Ac1-SST* transgenic lines were grown similarly. All T_4_ plants were grown under water-limiting field conditions. Three best performing lines (*Ac1-SST9*, *Ac1-SST26*, *Ac1-SST35*) were selected for further analysis. Homozygous plants of the T_5_-T_6_ generations were used for measurement of agronomic traits in the field. The T_6_ plants were used for molecular analyses, and also used for evaluation of photosynthetic fluorescence parameters in the field.

### Quantitative Reverse Transcription PCR and Southern Blot Analysis

The total RNA was extracted using the RNA purification kit (TIANGEN, Beijing, China) from leaf samples of T_5_ *Ac1-SST*-overexpressing cotton plants. The reverse transcription was performed using EasyScript One-Step gDNA Removal and cDNA Synthesis SuperMix (TRANSGEN, Beijing, China). The relative expression levels of *Ac1-SST* were quantified by quantitative reverse transcription PCR (qRT-PCR) using LightCycler^®^ 480 System (Roche Diagnostics Corporation, Indianapolis, IN, United States) and SYBR Premix Ex Taq II (TAKARA, Dalian, China). The primers used for qRT-PCR experiments are listed in Table 1. Cotton Ubiquitin7 (DQ116441.1) was used as an internal control. Each analysis was repeated three times using different samples. The relative expression levels were calculated using the 2^–ΔΔ^ ^CT^ method (Livak and Schmittgen, 2001).

Total DNA from cotton plants transformed with pBI121-*Ac1-SST* (T_7_ generation) was extracted using DNA Secure Plant Kit (TIANGEN, Beijing, China), and the presence of *Ac1-SST* gene was confirmed by genomic PCR using the primers in Table 1. About 15 μg of genomic DNA was digested with *Xba*I or *Sac*I for overnight to assure a complete digestion. The digested DNA was subjected to Southern-blot analysis as described by Liu et al. (2019).

### Drought Treatment in the Field

**FIGURE 1:**
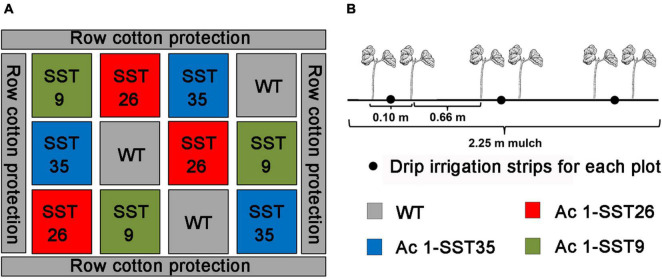
Experimental field design **(A)**, and *Ac1-SST* transgenic cotton and wild-type control plants planted in each plot. **(B)** To evaluate drought tolerance in the field. Planting distance between the two drip irrigation strips was 66 cm and the distance between two rows of cotton planted on each drip irrigation strip was 10 cm.

Drought treatments in the field were carried out in Shihezi, XinJiang, China (N44°20′,E85°30′) from April to October in 2019 and 2020. Shihezi area has a typical temperate continental arid climate with very little rainfall in summer (Table 2) where the relative soil water content can drop down to 11% in absence of irrigation (Zhang et al., 2016). Therefore, irrigation is necessary for agricultural production. In this case, the growth of cotton without irrigation was used as drought stress test. The seeds of the homozygous T_5_-T_6_ transgenic *Ac1-SST* cotton lines and the wild-type (R15) were sown in the field and were divided into two treatments. One treatment was watered regularly according to the rate needed for local agricultural production and this group was considered as control group. While for the other treatment, water was withheld for the rest of the growing season after seed germination where the plants only received natural precipitation and this group was considered as drought stress group. Drip irrigation under mulch was used for growing cotton in the present experiment. Three drip irrigation strips were covered with a 2.25-m land mulch. Two rows of cotton were planted on both sides of each drip irrigation belt. The inter-row spacing was 66 cm and interplant spacing within rows was 10 cm. The experiment was laid out in completely randomized block design with three replicates (Figure 1). The area for each plot was 13.5 m^2^ (2.25 m × 6 m), and each plot had about 300 plants. In each plot, 45 plants were randomly selected for agronomic trait analyses, and all plants in each replicate plot were included in yield analysis.

**TABLE 2:** Climate and irrigation conditions from April to September of 2019–2020.

Year	Average rainfall (mm/day)	Average relative humidity (%)	Average high temperature (°C/day)	Highest temperature recorded (°C)	Average temperature (°C/month)	Normal irrigation (m^3^/m^2^)	Drought irrigation^a^(m^3^/m^2^)
2019	0.85	39.2	27.9	38	21.1	0.45	0.032
2020	0.31	23.7	27.5	38	27.5	0.52	0.026

*^a^Plants were only irrigated for about 3 h after sowing.*

### Drought Treatment in the Greenhouse

In order to minimize stress and environmental variability, drought stress treatment under controlled conditions was performed to test the effects of *Ac1-SST* overexpression on drought tolerance. The seeds of T_6_ transgenic plants and the wild-type were sown in pots with a diameter of 15 cm and grown for about one and a half months in a greenhouse with 16/8 h light/dark photoperiod. The plants were watered every week to maintain optimal soil moisture levels. Then, plants were subjected to drought stress by withholding water for 32 days. Samples from the third leaves from the top were collected before drought stress and on after 32 days of drought stress. The leaves were immediately frozen in liquid nitrogen and stored at –80°C until used for measurement of physiological and biochemical parameters.

For measuring Ac1-SST enzyme activity, seeds of three transgenic lines along with the wild-type were soaked in water for overnight and then grown in flower pots. The plants were grown in a growth chamber with a 16-h light/8-h dark photo period and watered normally until the plants developed 1–2 leaves. Drought stress tests were then carried out. The experiment was divided into two parts; one group was not watered, and this group was considered as drought-stressed group. The other group was well-watered and was considered as control group. The leaf samples were collected after 16 days of treatment from both groups, and used for the measurement of Ac1-SST enzyme activity.

### Measurement of Physiological Parameters (Soluble Carbohydrates, Malondialdehyde, Proline, Relative Water Contents and Ac1-SST Enzyme Activity)

The leaves of T_6_ *Ac1-SST* transgenic lines and wild-type plants were sampled at the blooming and boll-bearing stages under the field and were used to measure soluble carbohydrates, proline, Malondialdehyde (MDA) content and relative water contents (RWC) as previously described (Liu et al., 2019).

The total soluble sugar contents were measured using the anthrone reagent (Dubois et al., 1951). Water soluble carbohydrates were determined according to the method of Zhang and Qu (2003). Leaf samples of the transgenic and wild-type plants were collected, freeze-dried, crushed into a powder, and passed through a 0.5 mm sieve. Samples of 0.2 g of the freeze-dried leaf material were added to 8 mL aliquots of 80% ethanol at 80°C followed by two extractions with 8 mL of double distilled water at 60°C, then the samples were cooled at room temperature and the extractions were used for measuring the total soluble sugars, sucrose, fructose, glucose, and 1-kestose. The samples were centrifuged at 4000 × *g* for 15 min, and the supernatants were passed through 0.2 μm filter membrane. The supernatants injection volume was about 3 mL. Total sugar, sucrose and fructose were quantified by measuring absorption at 620, 500, and 480 nm, respectively. To determine glucose contents, 4 mL of glucose reaction solution was added to 2 mL extraction mixture to start the reaction at 30°C for 5 min. Glucose reaction solution was prepared by mixing 10 mg of horseradish, 10 mg of *o*-dianisidine and 0.1 mL of glucose oxidase (1000 U/mL dissolved in acetic acid buffer with PH 5.5), and finally the volume of the reaction solution was made to 100 mL with water. Subsequently, the reaction was terminated with 8 mL of 10 mmol/L sulfuric acid. The glucose content was quantified by measuring the absorbance at 460 nm. The 1-kestose content was measured using High Performance Liquid Chromatography (HPLC) method.

The activity of Ac1-SST enzyme in the transgenic plants was measured in terms of the production of Ac1-SST enzyme product (kestose) per minute. Briefly, leaf samples (2 g) were homogenized in 10 mL distilled water; the homogenate was filtered through four layers of gauze and then centrifuged at 3500 rpm for 10 min at 4°C. The supernatant was used to measure the Ac1-SST enzyme activity. Aliquots of 10 mL of the crude enzyme extract was added to 10 mL sucrose solution (mass volume ratio of 10%) and the mixture was kept on an incubated shaker at 35°C and a speed of 200 rpm for 60 min. The reaction was terminated in a water bath at 85°C for 10 min. Then, the mixture was centrifuged at 12000 rpm for 2 min, and the supernatant was used to measure the Ac1-SST enzyme activity using HPLC (Dionex-3000, United States). One unit of Ac1-SST enzyme activity was defined as the amount of enzyme required to produce 1 μmol kestose min^–1^. The Ac1-SST activity was calculated using the formula: (2 × 1000 × GF_2_)/(0.504 × T × M_2_), where 2 was the total sugar content of 20 mL of 10% sucrose solution (g), GF_2_ was the percentage of kestose (%), 0.504 was the weight of 1 μmol kestose (mg), T was the time of reaction (min), and M_2_ was the fresh weight of leaf sample (g).

### Measurement of Agronomic Traits in the Field

The plant height, number of bolls and fruiting branches, and seed yield per plant of T_5_-T_6_ transgenic cotton and wild-type plants was recorded in 2019 and 2020. The agronomic traits were measured at the boll-bearing stage. In addition, to determine the actual cotton yield, all plants in each replicate plot were used for yield analysis.

### Measurement of Photosynthetic and Fluorescence Parameters in the Field

The photosynthetic and fluorescence parameters of T_6_ transgenic and wild-type plants were measured for the control and droughted plants in the field in the year 2020. The measurements of photosynthetic parameters (Pn and E) were performed on the fourth fully expanded leaf from the top of cotton plants in the morning between 9 and 11 AM using GFS3000 (WALZ, Germany). The irrigated plants (Normal group) were measured one week after watering. Chlorophyll fluorescence parameters (Fv/Fm, ΦPSII and PI_total_) were measured using a Mini-PAM light quantum analyzer (Zeal Quest Scientific Technology Co, Ltd, Germany) and ultra-portable Modulated Chlorophyll Fluorometer MINI-PAM (WLAZ). The water use efficiency (WUE) of cotton leaves was calculated as the photosynthetic index Pn/E (WUE = Pn/E).

### Statistical Analysis

All histograms were plotted using Origin 9.0 software (Origin Lab; Northampton, MA, United States). Data from this study were statistically analyzed by running one way ANOVA using SPASS 17.0. The least significant difference analysis was used to identify samples with significant differences (* Significant difference at *p* < 0.05, ^**^Significant difference at *p* < 0.01). Data are presented as the means ± SD of three independent replicates.

## Results

### Generation and Screening of Transgenic Cotton Plants

**FIGURE 2:**
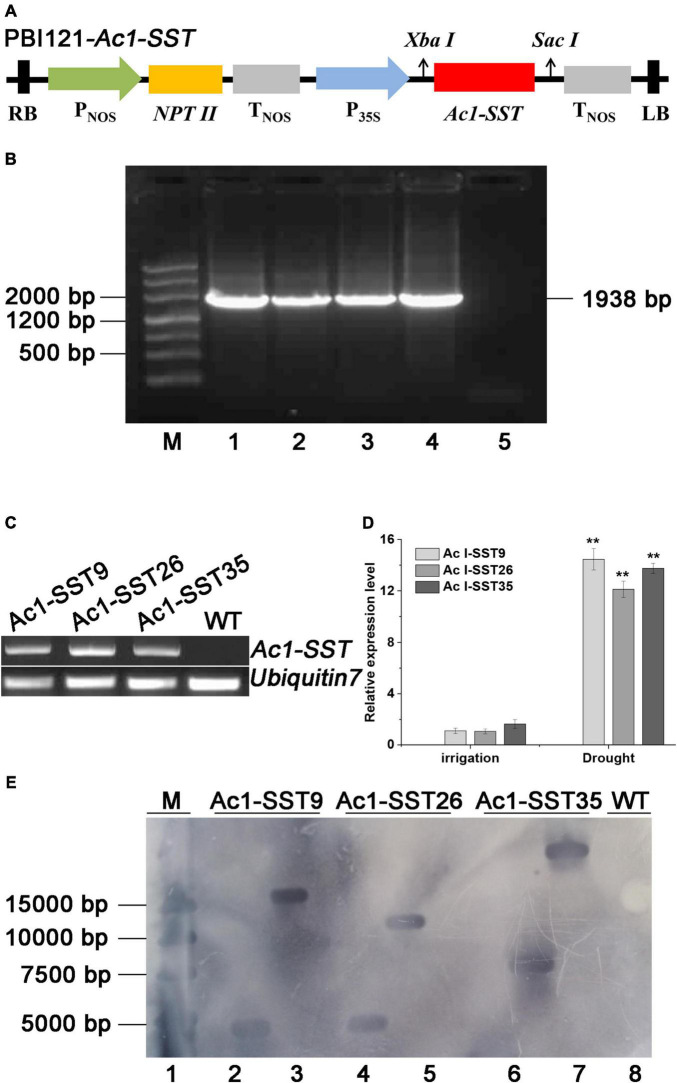
Stable inheritance and expression of *Ac1-SST* in the transgenic cotton lines **(A)**, schematic representation of the plant expression construct pBI-121-Ac1-SST. **LB**, left border; Pnos, promoter of nopaline synthase gene (nos); **Tnos**, terminator of nopaline synthase gene (nos); NPTII, kanamycin resistance gene; 35S, CaMV 35S promoter; RB, right border. **(B)** PCR identification of T_6_ transgenic *Ac1-SST* cotton plants. M, molecular Marker; 1, plasmid pBI-121- *Ac1-SST* DNA template was used as positive control; 2–4, DNA template of three transgenic *Ac1-SST* lines (9, 26, 35); 5, negative control without DNA template; **(C)** *Ac1-SST* reverse transcription PCR (RT-PCR) in the transgenic lines. Lanes: *Ac1-SST9*, *Ac1-SST26*, and *Ac1-SST35*, three transgenic cotton plants; WT, wild-type. Ubiquitin7 was used as an internal control. **(D)** Quantitative RT-PCR (qRT-PCR) analysis of *Ac1-SST* transgenic cotton lines. Relative expression levels were calculated by the 2^–△△^^CT^ method [△△CT = (C_T_,_Ac1–SST9/Ac1–SST26/Ac1–SST35_-C_T_,_UBQ_)-(C_T_,_Ac1–SST9/Ac1–SST26/Ac1–SST35_-C_T_,_UBQ_)_normal irrigation of Ac1–SST9_]. Error bars indicate standard deviation. Two Asterisks indicate significant differences between the transgenic and wild-type plants at *p* < 0.01. **(E)** Southern-blot analysis of the three T_6_ generation *Ac1-SST* transgenic cotton lines (9, 26, and 35). Line 1: Marker; Line 2, 4, and 6: genomic DNA of transgenic cotton lines cut by *Xba*I; line 3, 5, and 7: genomic DNA of transgenic cotton lines cut by *Sac*I, line 8: genomic DNA from wild-type R15 cut by *Sac*I. The digested DNA samples were separated on agarose gel, blotted onto nylon membrane and hybridized to digoxin labeled *Ac1-SST* probe as described in section “Materials and Methods”.

**FIGURE 3:**
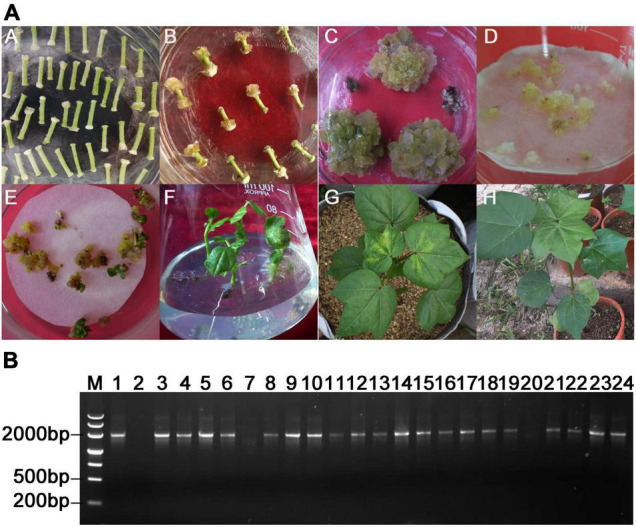
Acquisition of transgenic cotton plants and PCR identification of grafted plants from tissue culture seedlings of T_0_ generation **(A)**, Various stages of *Agrobacterium tumefaciens*-mediated transformation of cotton variety R15: co-culture, selection on kanamycin-containing medium, somatic embryo-genesis, generation of intact transgenic plant with normal shoot and roots **(A–F)**, Kanamycin resistance identification of T_0_ transgenic *Ac1-SST* transgenic grafted plants (**G:** positive plants, **H:** negative plants). **(B)** PCR identification of T_0_ transgenic *Ac1-SST* cotton plants M: Molecular Marker; 1: plasmid pBI-121- *Ac1-SST* DNA template was used as positive control; 2: Negative control without DNA template; 3–6, 8–19, and 21–24: PCR-positive transgenic *Ac1-SST* lines; 20: PCR-negative *Ac1-SST* lines.

To create *Ac1-SST* transgenic cotton, the *Ac1-SST* gene was cloned into binary expression vector pBI121 (Figure 2A). A total of 46 tissue culture seedlings were obtained via *Agrobacterium*-mediated transformation of cotton hypocotyls (Figure 3A). And T_0_ seedlings were grafted onto the stem of wild-type (R15) plants to ensure their survival and receive seeds. The 42 surviving plants were first sprayed with kanamycin to determine kanamycin resistance (Figures 3A–G,H), the 37 plants with kanamycin resistance (Figure 3A–H) were confirmed the target gene by genomic PCR (Some of the PCR results are shown in Figure 3B) and obtained T_1_ seeds. T_1_ generation seeds were planted for propagation in the field, the plants were sprayed with kanamycin and the presence of *Ac1-SST* gene was confirmed by genomic PCR. T_2_-T_6_ generations were obtained through the same methods as the T_1_ generation at each of the previous generations. All T_4_ generations was grown under water-limiting field conditions. Three best performing lines (*Ac1-SST9*, *Ac1-SST26*, *Ac1-SST35*) were selected for further analysis. *Ac1-SST* gene in three T_6_ generation transgenic lines was confirmed by PCR (Figure 2B) and semi-quantitative PCR (Figure 2C) and qRT-PCR (Figure 2D). The results confirmed the RNA expression of the *Ac1-SST* transgene in these three cotton lines both under drought stress and normal irrigated conditions. In addition, Southern-blot analysis of T_6_ generation of the transgenic lines showed a single T-DNA insertion in all selected lines (Figure 2E).

### *Ac1-SST*-Overexpressing Plants Showed Improved Drought Tolerance

**FIGURE 4:**
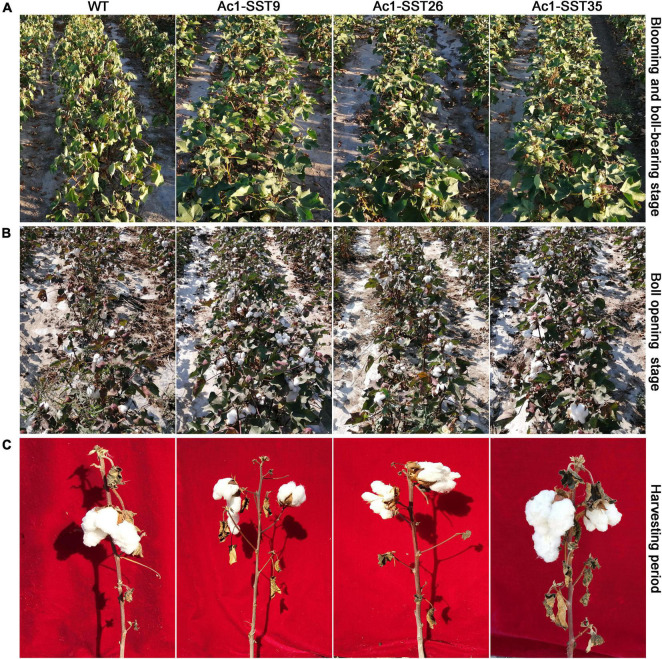
The experimental setup and rudimentary growth phenotypes of the wild-type and three *Ac1-SST* transgenic cotton lines grown in 2020. The plants were subjected to drought stress-treatments in the field as described in section “Materials and Methods” and pictures of the plants were taken in 65 and 130 days after planting. **(A)** Plants at vigorous growth, blooming and boll bearing stage; **(B)** plants at the boll opening stage; **(C)** individual plants at harvesting stage.

Under drought stress in the field, as shown in Figure 4, the growth of three T_6_ generation lines was significantly superior to that of wild-type at both blooming and boll-bearing, and boll opening stages. Especially in the blooming and boll-bearing stages, the leaves of the three transgenic lines remained turgid, while the leaves of wild-type plants showed wilting from top to bottom (Figure 4A). In the boll opening period, the number of opening bolls of the transgenic plants was significantly more than that of wild-type. In contrast, only few opening bolls were visible on the wild-type plants, and almost all of the leaves were shed (Figures 4B,C). However, under normal irrigation condition in the field, there is no significant difference between the transgenic and wild-type plants at different growth and development stages.

**FIGURE 5:**
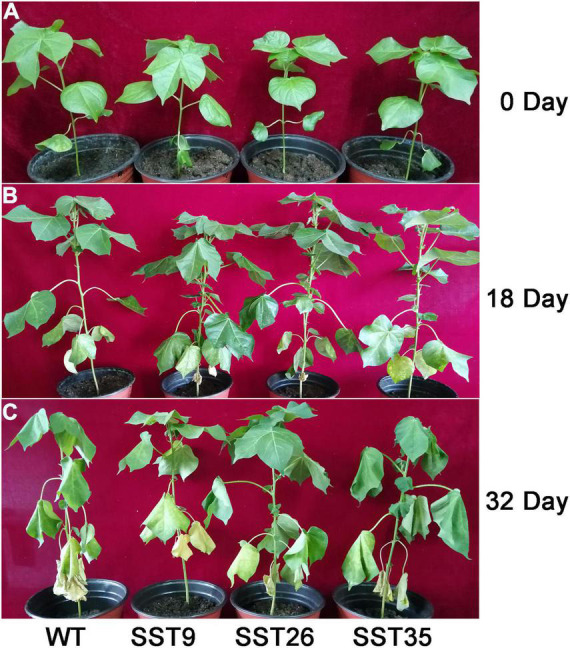
Phenotypic changes of the droughted wild-type and T_6_ plants grown under controlled greenhouse conditions. **(A)** Growth phenotype of T_6_ plants before drought stress. (0 day: Plants of wild-type and three transgenic *Ac1-SST* cotton lines (9, 26, and 35); **(B)** growth phenotype of T_6_ plants on the 18th day of drought treatment (18 day: 18th day of water withholding); **(C)** growth phenotype of T_6_ plants on the 32th day of drought treatment (32 day: 32th day of water withholding).

Under drought stress in the greenhouse, Figure 5A shows that prior to the drought-stress treatment, there was no significant difference between the transgenic lines and the wild-type. After 18 days of water withholding, the leaves of the transgenic and wild-type plants looked similar (Figure 5B). However, after 32 days of treatment, many of the wild-type leaves appeared severely wilting whereas the transgenic leaves showed only mild wilting (Figure 5C). Ectopic *Ac1-SST* expression improved the tolerance of cotton to drought stress.

### *Ac1-SST*-Overexpressing Plants Showed Enhanced Agronomic Traits in the Field Under Drought Stress

To quantify the drought tolerance of the three *Ac1-SST* transgenic lines, we also measured the agronomic traits of transgenic lines and the wild-type. As shown in Table 3, under well-watered condition in 2019, the three transgenic lines showed significantly enhanced agronomic traits including number of fruiting branches (16.2, 20.1, and 16.2%, respectively), boll number (21.4, 18.6, and 20.0%/plant, respectively), cotton seed yield (12.9, 10.3, and 12.1%/plant, respectively) compared with the wild-type. However, the plant height of three transgenic lines was slightly reduced compared to the wild-type. A similar trend was observed in 2020 for the transgenic lines under well-watered condition. However, under drought condition, the transgenic lines had significantly increased plant height, number of fruiting branches, number of bolls and seed yield compared with the wild-type (Table 3). In addition, the seed yield per plant of the transgenic lines increased by an average of 28.2 and 20.6% (Table 3) in both years. Data in Table 3 shows that the seed yield of the transgenic lines increased by 34.1 and 21.1%, as compared with that of wild-type plants in 2019 and 2020, respectively.

**TABLE 3:** Agronomic traits of the wild-type and three transgenic lines, grown under drought in the field.

Year	Gene lines	Plant height (cm)	Fruiting branch number (/plant)	Boll number (/plant)	Cotton seed yield (g/plant)	Cotton seed yield (kg/plot)
2019 Drought	WT	36.73 ± 2.57	1.73 ± 0.44	1.91 ± 0.29	5.48 ± 0.29**	1.24 ± 0.04
	Ac1-SST9	39.36 ± 1.36*	2.91 ± 0.51**	3.45 ± 0.50**	7.35 ± 0.46**	1.53 ± 0.07**
	Ac1-SST26	39.09 ± 1.36*	2.82 ± 0.39**	3.36 ± 0.77**	6.78* ± 0.38**	1.50 ± 0.08**
	Ac1-SST35	38.73 ± 3.54*	3.00 ± 0.74**	3.18 ± 0.72**	6.95 ± 0.44**	1.48 ± 0.06**
2019 Irrigation	WT	86.27 ± 4.41	6.18 ± 0.94	6.36 ± 1.07	25.74 ± 2.48	11.51 ± 0.76
	Ac1-SST9	83.64 ± 3.75	7.18 ± 1.03*	7.73 ± 0.86*	29.08 ± 2.84	12.72 ± 0.85*
	Ac1-SST26	82.36 ± 3.28	7.45 ± 1.08*	7.55 ± 1.44*	28.41 ± 2.72*	12.19 ± 0.68*
	Ac1-SST35	81.55 ± 2.43*	7.19 ± 0.72*	7.64 ± 1.72*	28.87 ± 2.41*	12.66 ± 0.72*
2020 Drought	WT	32.42 ± 4.75	1.08 ± 0.28	0.75 ± 0.43	3.44 ± 0.23	0.96 ± 0.05
	Ac1-SST9	36.42 ± 2.25*	1.75 ± 0.60*	1.92 ± 0.64**	4.17 ± 0.44**	1.17 ± 0.07**
	Ac1-SST26	36.25 ± 2.28*	1.83 ± 0.37**	1.33 ± 0.47*	4.25 ± 0.52**	1.16 ± 0.05**
	Ac1-SST35	36.58 ± 2.06*	1.92 ± 0.28**	1.25 ± 0.43*	4.03 ± 0.24**	1.14 ± 0.08*
2020 Irrigation	WT	85.36 ± 4.60	6.36 ± 0.98	6.73 ± 0.86	31.44 ± 2.56	12.43 ± 0.77
	Ac1-SST9	82.09 ± 3.32	7.91 ± 1.83*	8.36 ± 1.82*	35.18 ± 1.74*	13.34 ± 0.59*
	Ac1-SST26	79.91 ± 3.45*	7.73 ± 1.21*	7.82 ± 1.34*	34.02 ± 2.55*	13.34 ± 0.34*
	Ac1-SST35	82.27 ± 3.44	7.64 ± 1.77*	7.91 ± 1.38*	34.58 ± 1.71*	13.63 ± 0.23*

Plot size was 13.5 m^2^. Values are means ± SD (*p < 0.05; **p < 0.01).

### *Ac1-SST* Cotton Lines Had Higher Proline, MDA, RWC and Increased Soluble Carbohydrates Contents Under Drought Stress in the Field and Greenhouse

**FIGURE 6:**
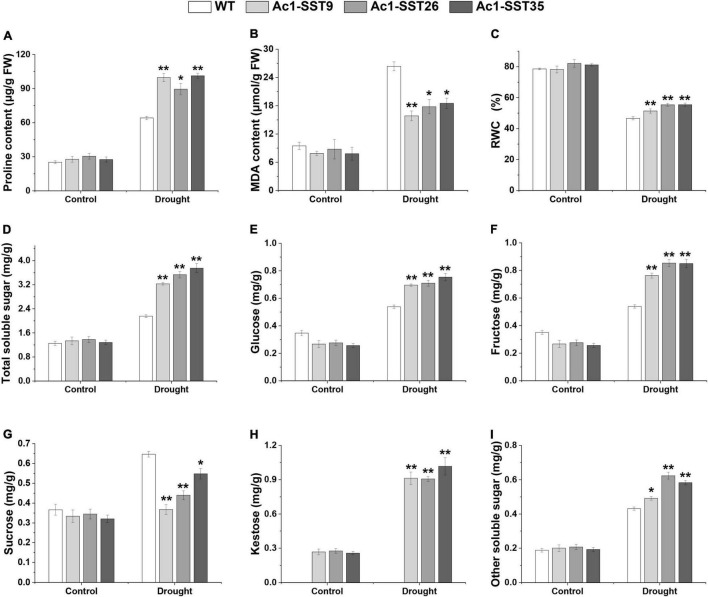
Physiological and biochemical analyses of *Ac1-SST* transgenic and wild-type plants under drought stress in the field. Control: normal precipitation and artificial irrigation throughout the growth period; Drought: natural precipitation after germination. **(A)** Proline content, **(B)** MDA content, **(C)** relative water content (%), **(D)** total soluble sugar content **(E)** glucose content, **(F)** Fructose content, **(G)** Sucrose content, **(H)** Kestose content, **(I)** other soluble sugars content. Error bars indicate standard deviation. *, In **(H)**, significant difference from transgenic lines with or without drought stress treatments; *, significant difference between the transgenic lines and wild-type control plants (Student’s *t*-test *p* < 0.05); **, significant difference between the transgenic lines and wild-type control plants (Student’s *t*-test p < 0.01); WT, wild-type (R15); *Ac1-SST*9, *Ac1-SST*26, and *Ac1-SST*35 indicate the three T_6_ transgenic lines.

**FIGURE 7:**
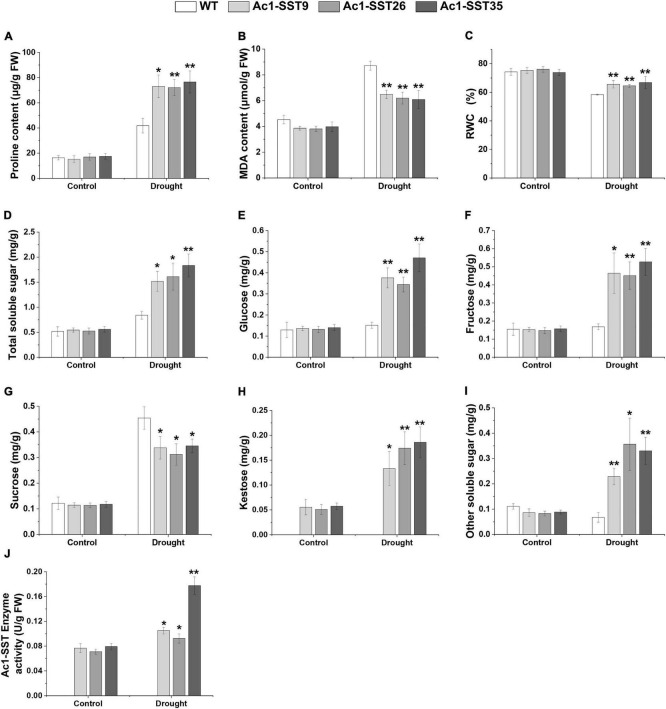
Physiological and biochemical analyses of *Ac1-SST* transgenic and wild-type plants with or without stress treatments under controlled greenhouse conditions Control: Well-watering; Drought: the leaves of 45 days-old plants with or without days drought treatments for 32 days. **(A)** Proline content, **(B)** MDA content, **(C)** relative water content (%), **(D)** total soluble sugar content, **(E)** glucose content, **(F)** fructose content, **(G)** sucrose content, **(H)** kestose content, **(I)** other soluble sugars content, **(J)** *Ac1-SST* enzyme activity of three independent biological repeats. Error bars indicate standard deviation. *, in **(H)**, significant difference from transgenic lines with or without drought stress treatments for 32 days; *, In **(J)**, significant difference from transgenic lines with or without drought stress treatments for 16 days; *, significant difference between the transgenic lines and wild-type control plants (Student’s t-test *p* < 0.05); **, significant difference between the transgenic line and wild-type control plants (Student’s *t*-test *p* < 0.01); WT, Wild-type (R15); *Ac1-SST*9, *Ac1-SST*26, and *Ac1-SST*35 indicate the three T_6_ transgenic lines.

Given that the transgenic plants have enhanced field phenotypes of agronomic traits, we also wanted to explore the physiological changes of the drought resistance phenotypes. Under drought stress in field, our results showed that both the *Ac1-SST* transgenic lines and wild-type had significantly increased proline content compared to the well-watered plants, but the transgenic lines had significantly higher proline contents compared with the wild-type (Figure 6A). MDA, a product of lipid peroxidation, was accumulated to lower levels in the transgenic cotton compared with the wild-type (Figure 6B). In addition, the transgenic cotton plants had a higher RWC than wild-type (Figure 6C). Similar results were obtained under greenhouse conditions (Figures 7A–C). However, under the well-watered conditions, the *Ac1-SST* transgenic lines and wild-type showed no significant differences both in the field and greenhouse.

Since the *Ac1-SST* gene is the key enzyme that initiates fructan synthesis (Gadegaard et al., 2008), we also measured the content of water soluble carbohydrates in both transgenic and wild-type cotton plants under the field and greenhouse conditions. As shown in Figures 6D–I, 7D–I, under drought stress condition, the total soluble sugars, glucose, fructose, sucrose, kestose, and other soluble sugar content significantly increased compared with the control. Except for sucrose, all water soluble carbohydrates in transgenic lines were significantly higher than in the wild-type. However, the greatest difference was that in the kestose content, which was only detected in the transgenic plants (Figures 6H, 7H), where it was significantly higher in droughted plants than that of control. These results are consistent with the higher transcription level of *Ac1-SST* gene and *Ac1-SST* enzyme activity under drought conditions (Figures 2C, 7J). Simultaneously, the sucrose content in transgenic cotton lines was significantly lower than in the wild-type (Figure 6D).

### *Ac1-SST* Cotton Lines Exhibited Improved Photosynthetic Capacity Under Drought Stress in the Field

**FIGURE 8:**
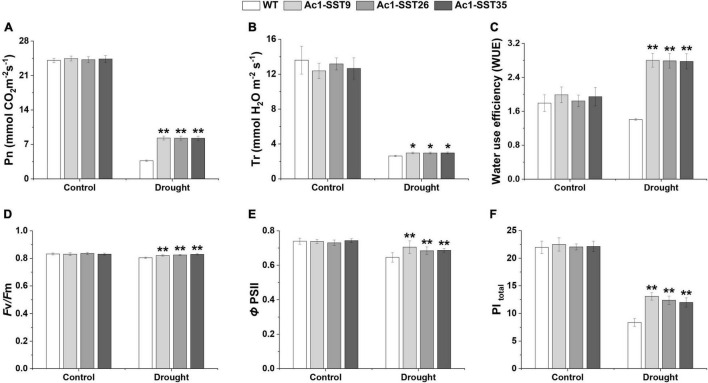
Photosynthetic performance of *Ac1-SST* transgenic and wild-type plants in the field experiments with or without drought stress treatments **(A)**, Net photosynthetic rate (Pn) **(B)**, Transpiration rate (E) **(C)**, Water use efficiency (WUE) **(D)**, Maximal PSII efficiency (Fv/Fm) **(E)**, Quantum yield of PSII electron transport (ΦPSII) **(F)**, the total performance index (PI_total_). Error bars indicate standard deviation. *, Significant difference between the transgenic line and wild-type control plants (Student’s *t*-test *p* < 0.05); **, significant difference between the transgenic lines and wild-type control plants (Student’s *t*-test *p* < 0.01); WT, wild-type (R15); *Ac1-SST*9, *Ac1-SST*26, and *Ac1-SST*35 indicate the three T_6_ transgenic lines.

Photosynthetic parameters such as net photosynthetic rate (Pn), Transpiration rate (E), WUE, optimal quantum yield of PSII (Fv/Fm), quantum yield of PSII electron transport (ΦPSII), and performance index total (PI_total_) were used to assess the photosynthetic performance of transgenic lines in the year of 2020. As shown in Figure 8, under normal irrigation condition (Control), all three transgenic line plants had relatively high Pn, E, Fv/Fm, ΦPSII, and PI_total_, and these metrics did not differ significantly between wild-type and transgenic plants. However, under drought stress condition, Pn, E, Fv/Fm, ΦPSII, and PI_total_ were significantly reduced in both wild-type and transgenic plants, but these were significantly higher in the transgenic lines. It is worth noting that compared with drought stress conditions; transgenic and wild-type plants had lower WUE under normal irrigation. Moreover, under drought stress, the WUE of the transgenic plants was significantly higher than that of wild-type. These data indicate that the transgenic lines had higher photosynthesis capacity and better PSII performance under the drought stress treatment in the field, thereby improves the drought tolerance as well as the agronomic traits and cotton seed yields of transgenic cotton.

## Discussion

Drought has adverse effects on crop biomass and yield. Therefore, improving crop drought-tolerance has been a long-standing goal to crop breeders. The application of biotechnology provides multiple possibilities for achieving this goal. According to previous studies, fructans can improve abiotic stresses tolerance, including drought, salt and freezing. Therefore, it is a feasible approach to improve the drought resistance of crop by introducing the genes related to fructans synthesis.

Drought can cause loss of the selective barrier function of cell membranes, leading to cell deformation and rupture, resulting in cell death. It has been shown that fructans can interact with cell membranes, thereby preventing lipid condensation and phase transitions, which may have a protective effect on water stressed plants (Demel et al., 1998; Vereyken et al., 2001). Fructans and fructan biosynthetic enzymes have been reported to be located in the vacuoles (Van Den Ende et al., 2005). However, fructans have been detected in the apoplast where their concentration was suggested to be controlled by differential leakage and/or fructan exohydrolase activity (Livingston and Henson, 1998). Moreover, Valluru et al. (2008) proposed a vesicle-mediated transport model for the movement of fructans from vacuolar to the apoplast, where fructans stabilize and protect the plasma membrane.

Hincha et al. (2002) compared the effects of fructans and glucans on cell membranes stability during air-drying, their results indicated that the accumulation of fructans with low degree of polymerization could play an important role in cellular dehydration tolerance. Subsequently, Hincha et al. (2007) compared the effects of fructan components in oats and rye on membrane stability during drying, and the results showed that it had optimal protective effects at degree of polymerization (DP) 4 and DP 3, DP 4, and DP 5, which further confirmed the above views. In our experiments, under the drought stress condition, the content of 1-kestose in the leaves of the transgenic lines was 3.6 and 2.5 times as much as that under normal irrigation under field and greenhouse conditions, respectively (Figures 6H, 7H). These results may also indicate that the increased drought resistance of transgenic plants under drought stress may be related to the accumulation of 1-kestose, which is a fructan with low DP. As a result, under drought stress in the field, the number of fruit branches and bolls of the transgenic plants increased by an average of 69 and 79.8%, respectively. However, other studies have shown that fructans with a high DP can directly interact with membranes (Demel et al., 1998; Vereyken et al., 2001), thereby stabilizing and protecting liposomes during freeze-drying and drying (Ozaki and Hayashi, 1996; Hincha et al., 2000, 2007). Therefore, fructans may have unique properties that would make them ideal solutes to stabilize plants cells under stress. Further research is needed to confirm this view.

Drought stress has negative effects on osmotic balance, and therefore, in order to mitigate this adverse effect, plants accumulate different organic and inorganic substances involved in osmotic adjustment, including sugars (glucose, sucrose, fructose, fructooligosaccharides, trehalose), sugar alcohols (mannitol), amino acids (proline, glycine) to reduce the osmotic potential, and improve cell water retention (Fang and Xiong, 2015; Singh et al., 2015). In this study, soluble carbohydrate and proline, two common compatible osmolytes with important roles in the oxidative stress responses of plants (Hare et al., 1998; Couée et al., 2006), were accumulated to significantly higher levels in the transgenic lines than in the wild-type plants after drought stress (Figures 6, 5B). Moreover, the sucrose content of transgenic lines was significantly lower than wild-types, the results suggest that sucrose has been converted into 1-kestose, and also that accumulation of greater sugar contents could result in lower intracellular osmotic potential, which contributed to the drought resistance in transgenic cotton lines. Moreover, the relative water content of transgenic lines leaves was significantly higher than that of wild-types (Figure 5C). Our results are consistent with earlier reports on transgenic tobacco plants expressing the *SacB* gene from *Bacillus subtilis*, which encodes sucrose-6-fructosyltransferase with subsequently improved drought tolerance (Ebskamp et al., 1994; Pilon-Smits et al., 1995). Ectopic expression of the three wheat genes *1-SST*, *6-SFT*, and *1-FFT* enhanced soluble fructooligosaccharide content and improved abiotic stress (drought, salt and low temperature) tolerance in tobacco plants (Bie et al., 2012). Another study also showed that drought-tolerant wheat varieties accumulated more soluble carbohydrates and had higher gene expression levels under drought conditions (Hou et al., 2018).

Drought stress is often accompanied by production of reactive oxygen species (ROS) (Apel and Hirt, 2004; Miller et al., 2010). However, excessive accumulation of ROS can cause cytotoxicity and membrane lipid peroxidation. Malondialdehye (MDA), is an indicator of membrane lipid peroxidation (Sharma et al., 2012). In the present study, the MDA contents of the transgenic cotton plants were significantly lower than that of wild-type when exposed to drought stress in the field, indicating that overexpression of *Ac1-SST* gene in cotton could effectively lead to avoidance of membrane lipid peroxidation and cytotoxicity.

Drought stress affects the rate of photosynthesis and consequently reduces growth and yield (Chaves et al., 2009). Higher photosynthesis rate is a desirable trait for plants, where it provides carbohydrates for plant growth and yield. Our study showed that drought stress reduced photosynthetic and fluorescence parameters in the leaves and much less decrease was found in the transgenic plants than in the wild-type, indicating that the transgenic plants had a higher photosynthetic efficiency than did the wild-type. This may be due to the improved osmotic balance ability of transgenic cotton plants to protect the photosynthetic system. In addition, ectopic *Ac1-SST* expression under drought conditions also resulted in higher leaf RWC, which may be the reason for the more vigorous growth and higher yield of transgenic plants.

## Conclusion

In general, we obtained stable inheritance of *Ac1-SST* in the transgenic cotton lines. Under drought stress in the field, compared with wild-type plants, the transgenic cotton plants showed increased tolerance to drought stress, and had lower MDA, higher soluble carbohydrate, proline contents as well as higher photosynthetic capacity. More importantly, the transgenic plants had higher expression levels of *Ac1-SST* and increased 1-kestose contents. This may also be the reason why transgenic plants had higher drought resistance, superior agronomic traits and higher seed yield under drought stressed in the field. This is the first report that transformation of the *Ac1-SST*, a key gene of fructan biosynthesis in cash crop cotton leads to improved drought tolerance and seed yield under drought stressed in the field.

## Data Availability Statement

The original contributions presented in the study are included in the article/supplementary material, further inquiries can be directed to the corresponding author/s.

## Author Contributions

JZ conceived the study and designed the experiments. RL and TJ designed and performed the experiments and wrote the manuscript. ZZ provided help with the cotton regeneration. ZY, ZL, SW, and HX provided the assistance with experiments. YL and AW provided vital advices on the manuscript. All the authors read and approved the final manuscript.

## Conflict of Interest

TJ was employed by Woda Agricultural Technology Co., Ltd. The remaining authors declare that the research was conducted in the absence of any commercial or financial relationships that could be construed as a potential conflict of interest.

## Publisher’s Note

All claims expressed in this article are solely those of the authors and do not necessarily represent those of their affiliated organizations, or those of the publisher, the editors and the reviewers. Any product that may be evaluated in this article, or claim that may be made by its manufacturer, is not guaranteed or endorsed by the publisher.
